# Experimental African trypanosome infection suppresses the development of multiple myeloma in mice by inducing intrinsic apoptosis of malignant plasma cells

**DOI:** 10.18632/oncotarget.18152

**Published:** 2017-05-24

**Authors:** Nathan De Beule, Eline Menu, Mathieu J.M. Bertrand, Mérédis Favreau, Elke De Bruyne, Ken Maes, Kim De Veirman, Magdalena Radwanska, Afshin Samali, Stefan Magez, Karin Vanderkerken, Carl De Trez

**Affiliations:** ^1^ Department of Hematology and Immunology-Myeloma Center Brussels, Vrije Universiteit Brussel, Brussels, Belgium; ^2^ Inflammation Research Center, VIB, Zwijnaarde-Ghent, Belgium; ^3^ Department of Biomedical Molecular Biology, Ghent University, Zwijnaarde-Ghent, Belgium; ^4^ Department of Molecular Immunology and Inflammation, VIB Inflammation Research Center, Ghent University, Ghent, Belgium; ^5^ Apoptosis Research Centre, NUI Galway, Ireland; ^6^ School of Natural Sciences, NUI Galway, Ireland; ^7^ Ghent University Global Campus, Yeonsu-Gu, Incheon, South Korea; ^8^ Department of Structural Biology Research Center (SBRC), Research Unit of Cellular and Molecular Immunology, Vrije Universiteit Brussel (VUB), Flanders Institute for Biotechnology (VIB), Brussels, Belgium

**Keywords:** multiple myeloma, T. brucei

## Abstract

Multiple myeloma (MM) is characterized by the accumulation of malignant plasma cells in the bone marrow (BM). Recently, several studies have highlighted the role of pathogens in either promoting or dampening malignancies of unrelated origin. *Trypanosoma brucei* is an extracellular protozoan parasite which causes sleeping sickness. Our group has previously demonstrated that trypanosome infection affects effector plasma B cells. Therefore, we hypothesized that *T. brucei* infection could have an impact on MM development. Using the immunocompetent 5T33MM model, we demonstrated a significant reduction in BM-plasmacytosis and M-protein levels in mice infected with *T. brucei*, resulting in an increased survival of these mice. Blocking IFNγ could only partially abrogate these effects, suggesting that other mechanisms are involved in the destruction of malignant plasma cells. We found that *T. brucei* induces intrinsic apoptosis of 5T33MM cells *in vivo*, and that this was associated with reduced endogenous unfolded protein response (UPR) activation. Interestingly, pharmacological inhibition of IRE1α and PERK was sufficient to induce apoptosis in these cells. Together, these results demonstrate that trypanosome infections can interfere with MM development by suppressing endogenous UPR activation and promoting intrinsic apoptosis.

## INTRODUCTION

Recently, an increasing number of publications have confirmed the positive or in rare cases the negative relationship existing between infectious agents and cancer. In fact, many bacteria or viruses were shown to exert a malignancy enhancing role. In 2012, a study published in Lancet attributed 16% of cancer occurring in 2008 to bacteria or viruses [1]. In Africa, this rate more than doubled as 36% of all cancers potentially result from infection [2]. Haematological malignancies, such as multiple myeloma (MM), have become a major cause of death and disability in developing countries and particularly in Sub-Saharan Africa, where it should start to slowly eclipse pathogenic diseases, such as HIV and malaria by 2030 [3].

MM is the 2^nd^ most frequent hematological cancer after non-Hodgkin lymphomas and represents 2% of all cancer related deaths in the USA with a median age at diagnosis of 69 years [4]. Due to the accumulation of malignant plasma cells, typical MM related end-organ damage can occur, such as anemia, renal failure, osteolytic lesions and immunosuppression [5].

African Trypanosomiasis (AT) is a chronic human and livestock infectious disease mediated by an extracellular protozoan parasite, *Trypanosoma*. The *in vivo* mouse model of AT is characterized by the development of an efficient type 1 inflammatory immune response mainly governed by the production of IFNɣ cytokine [6]. However, in order to establish a chronic infection within its host, AT have developed numerous mechanisms of immune escape [7]. For example, using an *in vivo* mouse model, our laboratory has demonstrated that *T. brucei* infections are able to modulate B cell homeostasis in primary and secondary lymphoid organs, respectively BM and spleen [8], hereby abolishing the vaccine-induced memory response against an unrelated pathogen [9] as well as blocking the development of arthritis in a collagen-induced arthritis mouse model [10].

Taken the fact that trypanosomes have adopted the capacity to induce a major remodeling of the host B cell compartment, we wished to investigate whether trypanosome infections could affect the outcome of MM. We herefore used the 5T33MM model, which is an immune competent model of MM, that mimics the human disease characteristics very closely in terms of BM homing, the production of M-protein (serum M-spike) and the development of osteolytic bone lesions [11].

## RESULTS

### *In vivo* effects of *T. brucei* infection in the 5T33MM murine model

**Figure 1:**
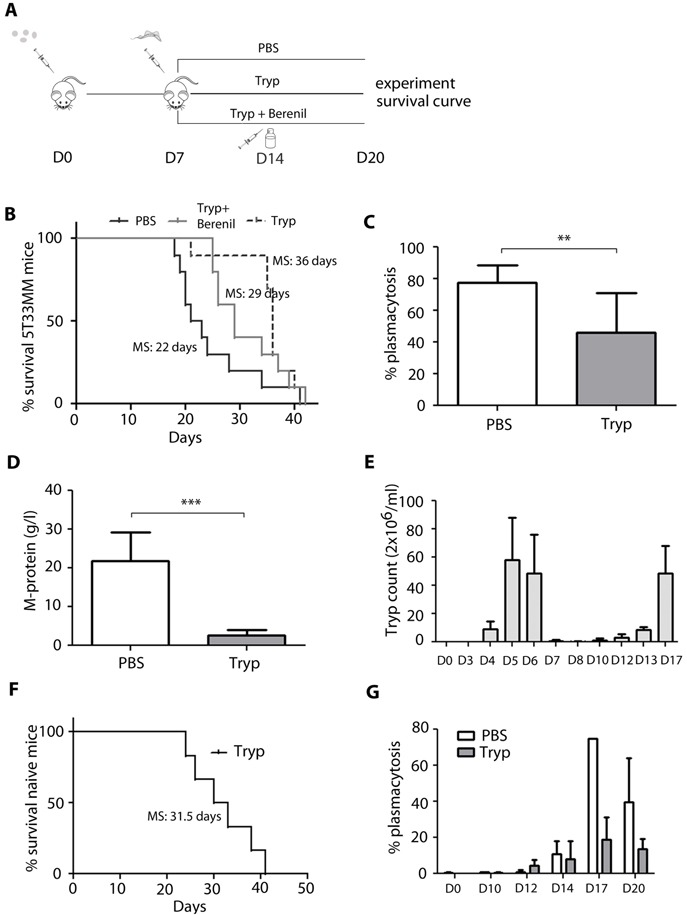
*In vivo* effects of *T. brucei* infection in the 5T33MM murine model **A**. Schematic of the experimental set-up. **B**.-**D**.+**G**. 7 days after inoculation with 5T33MM, mice were divided into 3 groups. One group was infected with *T*. *brucei*, one with *T. brucei* and Berenil, and one which served as control. **B**. Kaplan-Meier survival curve of 5T33MM diseased mice (*n* = 10/group) treated with or without *T. brucei* and Berenil. Survival of *T*. *brucei* infected mice was significantly longer compared to vehicle (*p* < 0,02). **C**.*-***D**. Tumor load was evaluated by measuring plasmacytosis in the bone marrow and M-protein levels in serum of all mice when the first mouse showed signs of morbidity (*n* = 10/group). **G**. Plasmacytosis was counted at different time points during MM development in treated and non-treated mice, 3 mice per time point. **E**.+**F**. naive C57BL/KaLwRij mice were infected with *T*. *brucei*. **E**. Parasitemia in blood was counted at different time points, 3 mice per time point. **F**. Kaplan-Meier curve of naive mice (*n* = 5). The bars represent mean +/− standard deviation (SD). *means *p* < 0,05, ** means *p* < 0,01, *** means *p* < 0,001.

Our group has previously shown that *T. brucei* infection is able to alter B cell homeostasis and their effector functions [8–10]. Therefore, we determined if trypanosome infection was affecting the development of the 5T33MM plasma cell malignancy in the C57BL/KalwRij mouse substrain; which is susceptible to hematological malignancies. In order to avoid any impact of the *T. brucei* parasite on the initial homing of the MM cells, 5T33MM cells were injected intravenously into C57BL/KalwRij mice one week prior to initiation of the trypanosome infection (Figure 1A). Interestingly we demonstrated that these mice lived significantly longer compared to uninfected mice administered only with 5T33MM cells (36 days *vs* 22 days, Figure 1B). In this malignant MM model, 18 to 22 days is the typical survival time post-5T33MM inoculation and this early morbidity is associated with substantial plasmacytosis in the BM and M-spike in the serum [11]. Therefore it is striking to note that *T*. *brucei* infection of MM mice, 7 days post-5T33MM administration significantly reduced BM plasmacytosis by 35% compared to uninfected 5T33MM mice twenty days after MM inoculation. This time point coincides with the moment that uninfected 5T33MM mice showed the first signs of morbidity (Figure 1C). This was also accompanied by a strong 85% reduction in serum M-spike (Figure 1D). These results most likely explain the extended survival of *T. brucei* infected compared to uninfected 5T33MM mice depicted in Figure 1B. Importantly, to date no information has been published on the evolution of parasitemia and survival of C57BL/KaLwRij mice infected with *T*. *brucei*. Hence, we observed that trypanosome infected C57BL/KaLwRij mice had a first peak of parasitemia at day 5-6 post-infection, which was followed by a second parasitemia peak at day 15 leading to morbidity due to uncontrolled parasitemia approximately 25 days post-infection onwards (Figure 1E and 1F). For this reason, it was technically not possible to follow the impact of trypanosome infection on the outcome of 5T33MM inoculation in susceptible C57BL/KaLwRij mice, beyond 4 weeks post infection (approximately 5 weeks post 5T33MM inoculation). Additional data showed that tumor suppression started 14 days post 5T33MM inoculation (7 days post *T. brucei* infection) (Figure 1G). Together, these results demonstrated that trypanosomiasis is significantly extending the survival of 5T33MM mice *via* the reduction of MM tumor load both in BM and serum. To determine whether the therapeutic effect on MM still persists upon control of infection, we treated mice receiving both MM and *T. brucei* with Berenil, which eradicates the parasites, one week after inoculation with *T. brucei*. This led to a median survival of 29 days which is 7 days longer than mice injected with MM alone but not as long as mice receiving both MM and *T. brucei* (Figure 1B). This indicates that the therapeutic effect does not persist upon parasite clearance but rather depends on the presence of live parasite to dampen tumoral load.

### Mechanisms of *T. brucei* mediated apoptosis of 5T33MM cells *in vivo*

**Figure 2:**
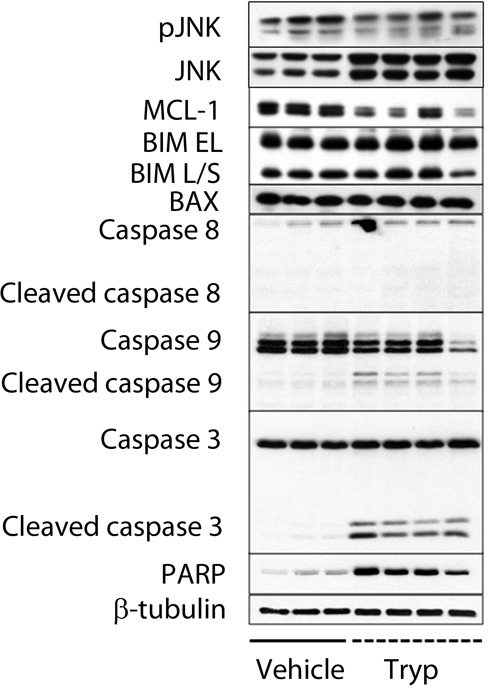
*T. brucei* mediated apoptosis of 5T33MM cells *in vivo* 7 days after inoculation with 5T33MM cells, mice were divided into 2 groups. One group was infected with *T*. *brucei (n = 12, pooled to 4 samples)*, the other served as control (*n* = 3). Mice were sacrificed at day 20. 5T33MM cells were isolated from the bone marrow and purified to > 85% by depletion of CD11b+ cells with MACS sorting. Western blot analysis for pJNK, JNK, MCL-1, Bim EL/L/S, Bax, caspase-8, caspase-9, caspase-3 and PARP was performed. β-tubulin served as loading control.

Next, the mechanisms implicated in *T*. *brucei* mediated tumor suppression were investigated. In the period between day 17 and 20 after 5T33MM inoculation, differences in bone marrow plasmacytosis between uninfected and infected 5T33MM mice were most pronounced (Figure 1G). Therefore day 20 was chosen as time point to isolate MM cells. We found that the MM cells died by apoptosis, as shown by cleavage of caspase-3 and PARP (Figure 2). Processing of caspase-9 but not of caspase-8 further demonstrated the activation of the intrinsic apoptotic pathways. Apoptosis induction was also associated with reduced MCL-1 levels, and normal expression of Bim and Bax. Although JNK levels were higher, the activated state of the kinase was unchanged (Figure 2).

**Figure 3:**
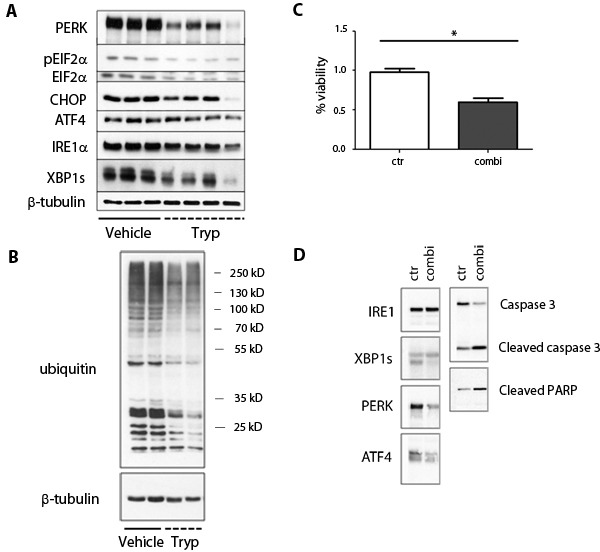
Effects of *T. brucei* on unfolded protein response pathways in 5T33MM cells **A.-B.** 7 days after inoculation with 5T33MM cells, mice were divided into 2 groups. One group was infected with *T*. *brucei (n = 12, pooled to 4 samples)*, the other served as control (*n* = 3). Mice were sacrificed at day 20. 5T33MM cells were isolated from the bone marrow and purified to > 85% by depletion of CD11b+ cells with MACS sorting. Western blot analysis for PERK, ATF4, CHOP, IRE1, GRP-78, caspase-12, pEIF2A, EIF2A, XBP-1 was performed. β-tubulin served as loading control. **B.** From 2 samples of each group as depicted in A, further analysis was performed for ubiquinated proteins. β-tubulin served as loading control. **C.** Viability assay was performed for 24h with MM cells (purity > 90%) of 3 different mice. Cells were treated with a combination of a PERK and IRE1α inhibitor. *means *p* < 0.05. **D.** One experiment representing 3 is shown of MM cells (purity > 90%) which were treated with the combination of PERK and IRE1α inhibitors for 21 hours, followed by western blot analysis.

In order to cope with the high demand of protein synthesis, highly secretory cells, such as plasma cells, constitutively activate the unfolded protein response (UPR), a signaling network initiated by IRE1α, PERK and ATF6 that aims at maintaining ER proteostasis [12]. Failure to activate the UPR in cells under ER stress increases the risk of misfolded proteins accumulation, which can result in cell demise by activation of the intrinsic apoptotic pathway [13]. Myeloma cells produce large amounts of immunoglobulins, and are therefore expected to rely on constitutive UPR activation for cell survival. We confirmed that the IRE1α and PERK branches of the UPR are constitutively activated in these cells by monitoring IRE1α-induced XBP1 splicing (XBP1s), PERK-mediated phosphorylation of EIF2α and expression of CHOP and ATF4 (Figure 3A). Interestingly, we observed that *T*. *brucei* infection reduced constitutive activation of IRE1α and PERK (Figure 3A) which led to a general reduction in the amount of ubiquitinated proteins (Figure 3B) and a reduction in the amount of secreted M-protein at early time points (data not shown). To determine whether reduction of UPR activation induced by *T*. *brucei* could decrease MM viability, we performed a viability assay using purified MM cells and UPR inhibitors. We found that the combination of a PERK inhibitor and an IRE1α inhibitor for 24 hours gave a significant 40% reduction in MM viability, indicating that blocking the IRE1α and PERK branches of the UPR induces MM cell death (Figure 3C). To further confirm the functionality of the UPR inhibitors, we treated MM cells with the combination of inhibitors and analyzed activation of the IRE1α and PERK pathways by western blot. We found a reduction in XBP1s, PERK and ATF4, which led to cleavage of caspase 3 and PARP (Figure 3D). Together, these results confirm that *T. brucei* infection reduces UPR activity and induces apoptosis of MM cells *via* the activation of the intrinsic caspase-9-dependent pathway, which ultimately leads to the cleavage of caspase-3 and PARP.

### Effect of anti-IFNɣ treatment on Trypanosoma mediated apoptosis *in vivo*

**Figure 4:**
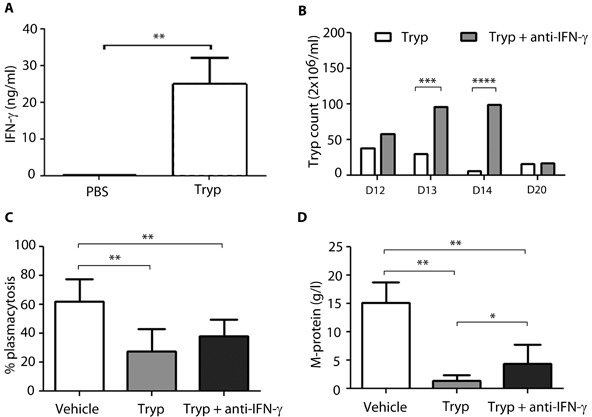
Effect of anti-IFNy treatment on Trypanosoma mediated apoptosis *in vivo* 7 days after inoculation with 5T33MMvv, mice were divided into 3 groups (*n* = 10/group): 2 groups were infected with *T*. *brucei*, one group served as control. Anti-IFNy treatment (300μg) was given to one of the *T*. *brucei* infected groups, 2-times a week intraperitoneally until the end of the experiment. All mice were sacrificed when first mice showed signs of morbidity. **A**. IFNy levels of *T. brucei* treated mice, measured in serum by ELISA. **B**. Blood parasitemia was counted at different time points. **C**.+**D**. Tumor load was evaluated by measuring plasmacytosis and M-protein levels. The bars represent mean +/− standard deviation (SD). *means *p* < 0,05, ** means *p* < 0,01, *** means *p* < 0,001, **** means *p* < 0,0001.

*T*. *brucei* infection in 5T33MM mice was accompanied by an increase in IFNy levels in serum up to 25 ng/ml at day 20 (Figure 4A). Recently, our group has underscored the detrimental role of IFNɣ in mediating the early loss of B cells by apoptosis during experimental African trypanosomiasis [14]. Therefore its potential role in the apoptosis of myeloma cells was investigated by treating *T*. *brucei* infected 5T33MM mice with anti-IFNɣ antibodies. Since the immune system no longer cleared the infection, this resulted in higher parasitemia serum levels at day 13 and day 14 (Figure 4B). Anti-IFNɣ treatment could only partially abrogate the effects on tumor load induced by *T*. *brucei* infection, namely an absolute increase in plasmacytosis of 10% (Figure 4C) and in M-spike of 3 g/l (Figure 4D). These data suggest that IFNɣ is involved but is not the only factor involved in *T*. *brucei* induced apoptosis of MM cells.

## DISCUSSION

The detrimental role of pathogens on the development of various cancers has been widely documented in recent years [1, 2]. Only in a small number of cases microbes were shown to play a positive role on cancer outcome [15, 16]. MM is the 2^nd^ most frequent hematological cancer, developing in the BM [5]. Although the survival of MM patients has been extended three-fold due to the introduction of new classes of anti-myeloma agents (e.g. proteasome inhibitors, immunomodulatory agents, histone deacetylase inhibitors and monoclonal antibodies,…) it still remains incurable for the majority of the patients [17–19]. So a better understanding of how MM cells proliferate, survive and resist therapy, is necessary to further develop new therapeutical agents.

The 5T33MM model is an immune competent model that mimics the human disease characteristics very closely after inoculation of C57BL/KaLwRij mice with 5T33MM cells. Therefore the impact of a parasitic infection on the development of MM was tested using an *in vivo* African trypanosomiasis mouse model. Results obtained show that 5T33MM mice infected with *T. brucei* parasites displayed a substantial decrease in M-protein level as well as a significant drop in plasmacytosis. As a consequence trypanosomiasis plays a surprisingly positive role on the outcome of myeloma by considerably prolonging the survival of infected mice compared to PBS treated counterparts. This result is rather unique as parasitic infections mainly promote the development of cancer [20]. For example, different species of parasites living in the water in regions in Middle East, Asia and Africa and causing *schistosomiases* can promote bladder and colorectal cancers [21–23]. It is important to note that towards the end of the experiments, *T. brucei* infected C57BL/KaLwRij mice were no longer dying from unrestrained MM cell proliferation but from uncontrolled parasitemia due to their increased susceptibility to African trypanosome infection compared to resistant C57BL/6 mice. Countering the infection by treating the *T. brucei* infected mice with Berenil resulted in reduced survival benefit indicating that the therapeutic effect depends on the presence of live parasite to dampen MM load. Hence, while exposure to trypanosome is obviously not a way forward in the search for a cure for MM, understanding the mechanisms that trypanosome exploits to alter the host’ B cell compartment might result in new insights that could fuel future anti-MM strategies.

Therefore, the mechanisms implicated in the suppression of MM cells following *T. brucei* infection were investigated. The analysis of multiple death-associated pathways by Western Blot has shown an upregulation of cleaved caspase-3 and PARP on enriched BM-derived 5T33MM cells from T. *brucei* infected compared to uninfected mice, demonstrating a higher rate of apoptosis. Previous results by our group have already put forward that trypanosome infection resulted in the induction of apoptosis by different subsets of splenic B cells, such as transitional, marginal zone and follicular B cells [8, 9]. The effect of *T. brucei* infection on marginal zone B cells has been linked to the upregulation of pro-apoptotic caspase-3 and the downregulation of anti-apoptotic Bcl-2 marker [9]. In this study, a reduction in the levels of anti-apoptotic MCL-1 was observed. More recently, our group has demonstrated that trypanosome can hamper the development of arthritis in DBA/1 prone mice by specifically targeting antigen specific antibody levels [10]. By investigating the upstream pathway leading to apoptosis, we found that MM cells isolated from infected mice displayed induced cleavage of caspase-9, indicating activation of the intrinsic apoptotic pathway, in which mitochondria play a crucial role. This is not unique to trypanosoma infection since infection of MM cells with myxoma virus also induces activation of caspase-9. However, in that case, caspase-9 activation resulted from increased caspase-8 cleavage which is a member of the extrinsic apoptosis pathway [24]. Moreover, we and others have demonstrated that chemotherapeutic drugs used as treatment in the context of MM, act *via* the activation of the intrinsic mitochondrial apoptotic pathway, usually in a p53-dependent manner [25, 26]. In order to elucidate which pathways could lead to caspase-9 cleavage we analyzed the role of the UPR in MM apoptosis. Due to an overactive ER, MM cells are particularly sensitive to UPR inhibitors [27]. Here we confirmed that Trypanosome infection reduces the UPR *in vivo* and that the 5TMM cells are sensitive to the combined use of a PERK and IRE1α-XBP1 inhibitor, leading to enhanced caspase-3 and PARP cleavage. This is particularly interesting since it has been demonstrated that activation of the UPR leads to drug resistance in MM cells [28]. By contrast, Leung-Hagesteijn et al have shown that MM cells arrested at the plasmablast level have low XBP1s levels and are thereby less responsive to treatment with the proteasome inhibitor Bortezomib [29]. The relationship between different pathogens and the UPR has already been investigated in several models, however results have been conflicting. It is generally assumed that bacteria and viruses activate the UPR in host cells, however it has been shown that S. negevensis suppresses the UPR [30]. Furthermore we cannot exclude that the UPR reduction is a secondary event to the apoptosis induction. To our knowledge, our paper is the first to highlight a role of *T. brucei* in the modulation of the UPR within its host during infection.

Finally, the role of IFNɣ was investigated as a candidate factor that might be implicated in the triggering of the intrinsic apoptotic pathway in MM cells. Indeed, IFNɣ has been shown to play a major role in anti-tumor responses as well as in the control of trypanosomiasis. *T. brucei* infections promote the development of a Th1-mediated immune response [31] and IFNɣ was recently implicated in the early B cell loss in a mouse model of African trypanosomiasis [14]. Our results showed that trypanosome infected mice treated with anti-IFNɣ blocking antibodies display statistically higher M-protein levels compared to untreated and infected mice, but no clear impact on BM plasmacytosis was seen. Since M-protein levels are the result of plasmacytosis both in BM and spleen, it is possible that IFNɣ mediated apoptosis of myeloma cells is more prominent in the spleen. However, both M-protein levels and plasmacytosis were still lower compared to uninfected 5T33MM mice. These data suggest that IFNɣ is partially implicated in the induced apoptosis of MM cells in *T. brucei* infected mice. Rakshit *et al*. suggested already a beneficial role for IFNɣ in MM. They demonstrated, using a solid Sp2/0 myeloma tumor that *Mycobacterium indicus pranii* induced anti-myeloma T cell responses, which were highly reduced in *Ifnɣ−/−* mice. However, no significant differences were observed in tumor progression, again suggesting a role for other mechanisms contributing to anti-tumor responses [32]. Together, our results demonstrate that African trypanosome infections are able to shortcut the development of MM in a mouse model, by UPR pathway reduction and the induction of malignant plasma cell apoptosis *via* an intrinsic caspase-9-dependent pathway leading to increased cleavage of caspase-3 and PARP. A thorough understanding of the fine-tuned molecular mechanisms implicated in this apoptotic process might potentially lead to the development of a new approach, strategy or therapy in order to fight MM.

## MATERIALS AND METHODS

### Mice and cell lines

C57BL/KaLwRijHsd mice were purchased from Envigo (Horst, the Netherlands). Housing and maintenance was done according to the regulations approved by the Ethical Committee for Animal Experiments, Vrije Universiteit Brussel (CEP n° 14-281-12). The 5T33MM mouse model used in these studies, was previously described [11]. 5T33MM cells were isolated from the BM of diseased mice by crushing hind legs and vertebrae. After red blood cell lysis, purity of > 90% MM cells was confirmed on May-Grünwald Giemsa stained cytospins. For western blot analysis and viability assays, a further purification was performed by depleting CD11b+ cells (cells of myeloid lineage) with MACS sorting (Miltenyi Biotech, Leiden, The Netherlands).

### Parasites and infection in mice

Clonal pleomorphic *T*. *brucei* AnTat 1.1E parasites were a kind gift from N. Van Meirvenne (Institute for Tropical Medicine, Belgium) and stored at −80°C. The pleomorphic AnTat1.1E (Eatro 1125 stock) *T. brucei* was used in this study as previously described [9]. Multi-wave parasitemia development characterizes this infection. Each wave represents a parasite population of different antigenic type. Each mouse was infected intraperitoneally with 5000 parasites 7 days after inoculation with 5T33MM cells. Where indicated, both infected and uninfected mice were treated i.p. with diminazene aceturate (Berenil or Veriben^®^, 40 mg/kg, CEVA SANTE ANIMALE, 33501, France) in PBS. Depending on the set-up of the experiment, parasitemia was evaluated by counting the number of parasites in the blood (diluted 1/200 in PBS) with a counting chamber and light microscope.

### ELISA

IFNɣ was quantified in the serum with an anti-IFNɣ ELISA (eBioscience, San Diego, CA, USA) following manufacturer's instructions.

### Viability assays

The effect of UPR inhibitors on MM viability was measured using a Cell Titer Glo Luminescent Viability assay (Promega, Madison, WI, USA). Briefly, purified MM cells were incubated with a PERK inhibitor (AMG PERK44, Tocris Bioscience, UK) and an IRE1 inhibitor (MKC8866). Inhibitors were used at 2.5μM and 10μM respectively, DMSO was used as control.

### Western blot

Western blot was performed on purified 5T33MM cells as described previously [12] using the following antibodies: pJNK (Thr183/Tyr185), JNK, MCL-1, Bim (EL/L/S), Bax, Caspase-8, Caspase-9, Caspase-3, PARP, ATF4, Caspase-12, XBP1, CHOP and β-tubulin, all purchased from Cell Signaling Technology (Boston, MA, USA). PERK, IRE1, GRP-78, pEIF2A (Ser52) and EIF2A were purchased from Santa Cruz Biotechnology Inc. (Dallas, TX, USA) and Ubiquitin from Enzo Life Sciences (Farmingdale, NY, USA).

### *In vivo* treatment with anti-IFNy

Mice were divided into 3 groups (*n* = 10/group): 2 groups were infected with T. *brucei*, one group served as control. Anti-IFNɣ (clone XMG1.2) and its isotype control rat IgG1 (clone HRPN) were purchased from Bioxcell (West Lebanon, NH, USA) and dissolved in PBS (Lonza, Basel, Switzerland). Anti-IFNɣ treatment (300μg/mouse) was given intraperitoneally to one of the T. *brucei* infected groups starting 2 days post infection. Injections were repeated 2-times a week until the end of the experiment. All mice were sacrificed when first mice showed signs of morbidity or paralysis.

### Statistics

Statistical analysis was done using GraphPad Prism 6.0 software. All data represent the mean ± standard deviation (SD). The Mann-Whitney U test was used to analyze the data. Kaplan-Meier curves were analyzed by means of the Log-Rank method. *p* < 0,05 (*), *p* < 0,01 (**), *p* < 0,001 (***), *p* < 0,0001 (****) were considered statistically significant.
