# Correction: Experimental African trypanosome infection suppresses the development of multiple myeloma in mice by inducing intrinsic apoptosis of malignant plasma cells

**DOI:** 10.18632/oncotarget.25336

**Published:** 2018-04-27

**Authors:** Nathan De Beule, Eline Menu, Mathieu J.M. Bertrand, Mérédis Favreau, Elke De Bruyne, Ken Maes, Kim De Veirman, Magdalena Radwanska, Afshin Samali, Stefan Magez, Karin Vanderkerken, Carl De Trez

**Affiliations:** ^1^ Department of Hematology and Immunology-Myeloma Center Brussels, Vrije Universiteit Brussel, Brussels, Belgium; ^2^ Inflammation Research Center, VIB, Zwijnaarde-Ghent, Belgium; ^3^ Department of Biomedical Molecular Biology, Ghent University, Zwijnaarde-Ghent, Belgium; ^4^ Department of Molecular Immunology and Inflammation, VIB Inflammation Research Center, Ghent University, Ghent, Belgium; ^5^ Apoptosis Research Centre, NUI Galway, Ireland; ^6^ School of Natural Sciences, NUI Galway, Ireland; ^7^ Ghent University Global Campus, Yeonsu-Gu, Incheon, South Korea; ^8^ Department of Structural Biology Research Center (SBRC), Research Unit of Cellular and Molecular Immunology, Vrije Universiteit Brussel (VUB), Flanders Institute for Biotechnology (VIB), Brussels, Belgium; ^9^ Department of Internal Medicine, Ghent University, Belgium

**This article has been corrected:** The correct author affiliation is given below:

**Mérédis Favreau^1,4,9^ and Magdalena Radwanska^3,4^**

^9^ Department of Internal Medicine, Ghent University, Belgium

Original article: Oncotarget. 2017; 8:52016-52025. https://doi.org/10.18632/oncotarget.18152

